# Circulating soluble CXCR4 fails to differentiate subtypes of primary aldosteronism

**DOI:** 10.1210/jendso/bvag048

**Published:** 2026-03-02

**Authors:** Wanying Chao, Yan Ren, Mingxi Zou, Lu Tan, Tao Chen

**Affiliations:** Department of Endocrinology and Metabolism, Adrenal Center, West China Hospital of Sichuan University, Sichuan 610041, P. R. China; Department of Endocrinology and Metabolism, Adrenal Center, West China Hospital of Sichuan University, Sichuan 610041, P. R. China; Department of Endocrinology and Metabolism, Adrenal Center, West China Hospital of Sichuan University, Sichuan 610041, P. R. China; Department of Endocrinology and Metabolism, Adrenal Center, West China Hospital of Sichuan University, Sichuan 610041, P. R. China; Department of Endocrinology and Metabolism, Adrenal Center, West China Hospital of Sichuan University, Sichuan 610041, P. R. China

**Keywords:** primary aldosteronism, CXCR4, soluble biomarkers, aldosterone-producing adenoma, molecular imaging

## Abstract

Accurate subtyping of primary aldosteronism (PA) is essential for treatment selection. This study examined whether soluble CXC chemokine receptor type 4 (sCXCR4) in peripheral blood reflects adrenal CXCR4 expression assessed by ^68^Ga-pentixafor PET/MR, and whether sCXCR4 aids PA classification. A total of 160 subjects were enrolled, including 88 patients with PA (52 aldosterone-producing adenoma [APA], 36 idiopathic hyperaldosteronism [IHA]) and 72 healthy volunteers. Serum sCXCR4 was measured by enzyme-linked immunosorbent assay. Group comparisons, correlation analyses, and receiver operating characteristic curves were used to evaluate diagnostic performance. Serum sCXCR4 did not differ significantly among APA, IHA, and controls, and showed no correlation with adrenal SUV_max on PET/MR. The discriminatory value for distinguishing APA from IHA was poor (AUC 0.52; 95% CI 0.40-0.64). These results suggest that circulating sCXCR4 does not mirror adrenal CXCR4 expression and has no diagnostic role in PA subtyping.

In 2018, Heinze et al first reported that CXC chemokine receptor type 4 (CXCR4) is highly expressed in 71% of APAs and 26% of cortisol producing adenomas (CPAs), and correlates closely with aldosterone synthase (CYP11B2) expression [2]. Their findings suggested that positron emission tomography/computed tomography using the CXCR4-specific ligand ^68^Ga-pentixafor (^68^Ga-pentixafor PET/CT) could noninvasively localize aldosterone-producing lesions [2]. Subsequent studies across multiple centers have validated these observations, supporting the utility of ^68^Ga-pentixafor PET/CT as a promising noninvasive tool for APA identification [3].

CXCR4 is a chemokine receptor that binds specifically to CXCL12, mediating key biological processes such as cell migration, recruitment, mobilization, and proliferation in both hematopoietic and nonhematopoietic tissues [4]. Soluble CXCR4 (sCXCR4), a nonmembrane form of the receptor potentially generated via proteolytic cleavage or alternative splicing, has been firstly detected in human serum in 2011 [5], and then this observation had been confirmed by several other studies [6, 7]. Elevated sCXCR4 levels have been reported in patients with inflammatory bowel disease, was as significant predictor of pancreatic cancer [6], and associated with poor prognosis in metastatic or recurrent colorectal cancer [7].

These observations raise the possibility that sCXCR4 could serve as a novel biomarker in clinical practice. However, to our knowledge, no prior studies have investigated serum sCXCR4 levels in PA or assessed its potential utility in subtyping APA and IHA.

To address this gap, we conducted a retrospective study enrolling 88 patients with confirmed PA (52 with APA and 36 with IHA) and 72 healthy volunteers (HVs) (Fig. 1A). The clinical characteristics of the participants were summarized in Table 1. PA diagnosis followed the Endocrine Society Clinical Practice Guidelines [1], requiring a positive plasma aldosterone (ng/dL)-to-renin (mU/L) ratio (ARR ≥ 3.7) and at least one positive confirmatory test, such as the captopril challenge test (CCT) and/or saline infusion test (SIT) demonstrating inadequate aldosterone suppression (plasma aldosterone concentration [PAC] <30% reduction 2 h after CCT, or PAC remained > 10 ng/dL after SIT). All patients underwent ^68^Ga-pentixafor PET/magnetic resonance imaging (PET/MR), and subtyping criteria for unilateral PA were defined as previously described [8].

**Figure 1:**
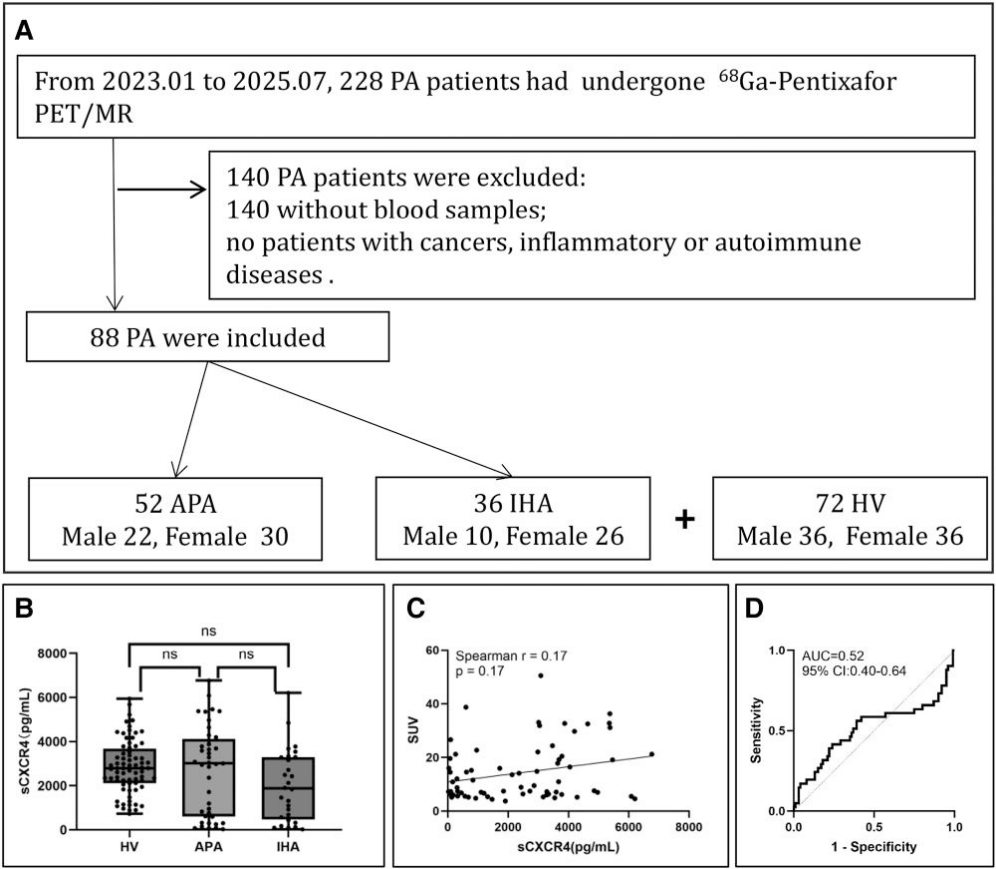
Measurement of sCXCR4 in patients with PA and evaluated its value in PA subtyping. (A) Flow chart of inclusion of patients with PA. Cortisol co-secretion had been excluded before ^68^Ga-Pentixafor PET/MR. (B) sCXCR4 levels in peripheral circulation. No significant differences were observed among patients with APA, patients with IHA, and HV. (C) Correlation analysis of sCXCR4 levels in peripheral circulation and CXCR4 expression level as indicated by ^68^Ga-pextixafor PET/MR in all patients with PA. For patients with unilateral lesions, the SUVmax of the typical adrenal nodule was used. For patients with bilateral adrenal lesions (including multiple nodules) or without an apparent lesion, the SUVmax among all detectable adrenal nodules or adrenal glands was used for the analysis. No significant relation was found (*r* = 0.17, *P* = .17). (D) ROC curve of sCXCR4 for PA subtyping. The results showed that sCXCR4 had poor value in differentiating APA from IHA.

**Table 1:** Clinical characteristics of patients with APA, IHA and heathy volunteers

	HV	IHA	APA
n.	72	36	52
Female n. (%)	36 (50.0)	26 (72.2)	29 (55.8)
Age	46.4 ± 14.6	52.1 ± 6.2	47.8 ± 7.4
Height (cm)	164.2 ± 7.5	161.8 ± 7.8	162.5 ± 10.1
Weight (kg)	63.3 (12.8)	70.0 (28.3)	71.5 (41.5)
BMI (kg/m^2^)	24.0 ± 3.2	27.0 ± 5.1	25.9 ± 4.9
W.C. (cm)	80.3 ± 9.4	92.8 ± 12.8	82.8 ± 15.2
SBP (mmHg)	119.3 (32.1)	150.0 (32.8)^†^	156.5 (23.8)^†^
DBP (mmHg)	84.3 ± 12.6	99.2 ± 14.8	105.5 ± 8.9
Hypokalemia (%)	0 (0.0)	21 (58.3)^†^	40 (76.9)^†^
K^+^ (mmol/L)	4.05 (0.33)	3.47 (0.52)^†^	3.27 (0.90)^†^
DRC (μIU/mL)	18.63 (17)	4.53 (14)^†^	5.54 (9)^†^
PAC (ng/dL)	11.75 (6.94)	24.70 (15.70)^†^	42.25 (49.65)^†‡^
ARR	0.71 (0.57)	3.67(5.91)^†^	14.10 (61.78)^†^
Adrenal lesions *			
Left n. (%)	0 (0.0)	19 (46.3)	23 (44.2)
Right n. (%)	0 (0.0)	1 (2.8)	28 (53.8)
Bilateral n. (%)	0 (0.0)	14 (38.9)	1 (1.9)
Normal n. (%)	72 (100.0)	2 (5.6)	0 (0.0)

Abbreviations: ARR, aldosterone-to-renin ratio; BMI, body mass index; DBP, diastolic blood pressure; DRC, direct renin concentration; PAC, plasma aldosterone concentration; SBP, systolic blood pressure; W.C., waist circumference.

Note: Continuous variables with a normal or approximately normal distribution (eg, age, height, BMI, waist circumference, and DBP) are presented as mean ± standard error. Variables with a non-normal distribution (eg, weight, SBP, serum potassium, DRC, PAC, and ARR) are presented as median (interquartile range). Categorical variables were compared using the chi-square test, and continuous variables were analyzed using one-way analysis of variance (ANOVA). All *P* values were two-tailed, and *P* < .05 was considered statistically significant. † *P* < .05 vs HV. ‡ *P* < .05 vs IHA. * *P* < .05 among the three groups.

HVs were recruited from an ongoing adrenal steroid profiling survey conducted in the Chengdu region of China. None of the participants had a history of hypertension, malignancy, malnutrition, adrenal disorders, or inflammatory diseases, nor had they used glucocorticoids within the preceding 3 months (Fig. 1A). Blood samples were collected, serum was isolated using standard procedures, and samples were stored at −80 °C for no longer than three years prior to analysis, without repeated freeze–thaw cycles. Serum sCXCR4 concentrations were measured using a commercially available ELISA kit (FineTest, Wuhan, China; Cat. No. EH2136; RRID: AB_3720204), under standard ELISA conditions without detergent treatment, vesicle disruption, or extracellular vesicle isolation. Under these conditions, the assay predominantly detects circulating sCXCR4. The study was approved by the Biomedical Ethics Review Committee of West China Hospital, Sichuan University (Approval No. 2019-556) and adhered to the principles of the Declaration of Helsinki. Informed consent was obtained from all participants.

Our results showed that serum sCXCR4 levels did not differ significantly among patients with APA, IHA, and HVs (*P* > .05) (Fig. 1B). Furthermore, serum sCXCR4 concentrations were not correlated with adrenal CXCR4 expression as assessed by PET/MRI Standardized Uptake Value (SUV)\_max values (SUV_max, Spearman's *r* = 0.17, *P* = .17) (Fig. 1C). Receiver operating characteristic (ROC) analysis revealed no diagnostic value of serum sCXCR4 in differentiating APA from IHA (area under the curve [AUC] = 0.52; 95% CI, 0.40-0.64) (Fig. 1D). These findings suggest that serum sCXCR4 does not serve as a useful biomarker for PA subtyping.

One possible explanation for these negative findings is the complex regulation of sCXCR4 production. sCXCR4 is expressed by multiple tissues and is modulated by various physiological and pathological factors, including inflammatory and neoplastic processes [9], which could obscure any specific association between serum sCXCR4 levels and adrenal aldosterone production.

Besides, we noticed that the sCXCR4 levels demonstrate substantial inter-individual variability within the APA group. Such heterogeneity may reflect differences in clinical or biological characteristics, including age, sex, adiposity, and renal function. To further explore this, we conducted subgroup analyses according to age (≥46.4 vs <46.4 years (mean age)), sex, BMI (≥24.0 vs <24.0 (mean BMI)), eGFR categories (≥90, 60-89, and 30-59 mL/min/1.73 m²), and the presence or absence of hypokalemia. No significant differences in sCXCR4 concentrations were observed across these subgroups, suggesting that additional unrecognized factors may contribute to the observed variability in APA. Another potential explanation relates to sample storage duration. Serum samples from APA patients were stored at −80 °C for a longer period than those from HVs (<3 years vs <6 months), and prolonged storage may have contributed to partial sCXCR4 degradation. Future studies using freshly collected serum samples are warranted to clarify this issue.

This study has several limitations. First, only one commercial ELISA kit was used to measure sCXCR4 levels. As sCXCR4 is a less well-characterized variant of the membrane-bound receptor, and given the lack of standardization across available detection assays, the validity of our measurements remains uncertain. However, the standard curve of this ELISA Kit demonstrated excellent performance, with a correlation coefficient of *r* = 0.986. Besides, the ELISA kits used in our study have been validated and applied in several independent investigations. For example, they have been used to measure plasma levels in patients with lumbar disc herniation and healthy controls [10], serum levels in patients with breast cancer [11], sCXCR4 concentrations in the culture supernatant of Caki renal cancer cells [11, 12], and in the supernatant of PC12 neuronal cells used as an experimental model for ocular diseases [13]. Notably, the serum sCXCR4 concentrations reported in two of these studies demonstrated good consistency between case and control groups, supporting the reliability of the assay [10, 11]. The second limitation is the relatively small sample size, which limits the generalizability of our findings.

In conclusion, although adrenal CXCR4-targeted molecular imaging appears promising for PA subtyping, measurement of serum sCXCR4 does not provide diagnostic value in this context.

## Data Availability

All datasets generated during and/or analyzed during the current study are not publicly available but are available from the corresponding author on reasonable request.
